# Variation in Quality of Women’s Health Topic Information from Systematic Internet Searches

**DOI:** 10.3390/healthcare13192537

**Published:** 2025-10-08

**Authors:** Bianca Kyrie Wanamaker, Ashley N. Tomlinson, Alivia R. Abernathy, Vanessa Cordova, Anika D. Baloun, Benjamin D. Duval

**Affiliations:** Biology Department, New Mexico Institute of Mining and Technology, Socorro, NM 87801, USA; bianca.wannamaker@nmt.edu (B.K.W.); ashley.bradshaw@student.nmt.edu (A.N.T.); alivia.abernathy@student.nmt.edu (A.R.A.); vanessa.cordova@student.nmt.edu (V.C.); anika.baloun@student.nmt.edu (A.D.B.)

**Keywords:** Google, internet, self-diagnosis, systematic search, women’s health

## Abstract

**Background/Objectives**: The internet has unquestionably altered how people acquire health information. Instead of consulting with a medical professional, billions of pages of information can be accessed by anyone with a smartphone. Women’s health issues have been historically and culturally taboo in many cultures globally; therefore, internet searches may be particularly useful when researching these topics. **Methods**: As an exercise in scientific information evaluation, we chose 12 non-cancer topics specific to women’s health and developed a scoring metric based on quantifiable webpage attributes to answer: What topics generate the highest and lowest scores? Does the quality of information (mean score) vary across topics? Does the variation (score deviation) differ among topics? Data were collected following systematic searches after filtering with advanced features of Google and analyzed in a Bayesian framework. **Results**: The mean score per topic was significantly correlated with the number of sources cited within an article. There were significant differences in the quality scores across topics; “pregnancy” and “sleep” scored the highest and had more sources cited per page than all other topics. The greatest variation in scores were for “cortisol” and “weight”. **Conclusions**: A purposeful, systematic internet search of 12 critical women’s health topics suggests that scrutiny is necessary when this information is obtained by a typical internet user. Future work should include review by medical professionals based on their interaction with patients who self-report what they know or think about a condition they present and respect, while educating, patients’ own internet searching.

## 1. Introduction

The development of the internet over the past 50 years has unquestionably led to a global democratization of information acquisition in the healthcare realm [1], while important concerns remain about the quality of that information [2]. Medical knowledge is arguably one of the most important areas for this phenomenon, as much of this information would have historically been held by relatively few medical professionals or in repositories and libraries not easily accessible to laypeople. Thus, ease of access to medical information is generally helpful to people with limited financial resources and in areas with limited access to healthcare. However, there is massive variation in individuals’ facility with searching for information, which may cause undue harm when search topics are of a perceived sensitive nature [3,4]. As women’s health issues have historically received less attention than conditions also or exclusively affecting men, and are more likely to be culturally taboo [5], internet searching is a viable option for obtaining information with some degree of anonymity. Here we give a brief background of the pros and cons of accessing women’s health information from the internet, then describe the methodology used to assess the medical veracity of webpages encountered following a systematic search. Unless otherwise stated, examples of pros and cons are considered within the contemporaneous context of the United States of America.

### 1.1. Why May Women Use the Internet Instead of Speaking with a Doctor?

First, many women who consult internet sources are indeed speaking with a medical professional as well, and finding medically accurate sources is still applicable to those individuals. However, it is worth considering factors that lead to reliance on internet sources as more than just supplemental data. For example, scheduling medical appointments can be burdensome, and even short wait times to see a healthcare professional are vastly longer than the few seconds it takes to access information via the Internet [6,7]. Other considerations include the following factors.

#### 1.1.1. Financial Cost

The internet allows millions of individuals to search for information regarding symptoms, treatment, and diagnoses for any disease or condition. Within the past 25 years, the internet has become a more accessible way to seek medical advice and information. It has been a useful tool for many communities that are not given the same medical opportunities as others, and it is often relied upon for supplemental medical information. There are a multitude of benefits that come from this information being so accessible to the public, one being the ability to gain information without direct monetary cost. The drastic increase in the cost of healthcare in the past several years has heightened internet usage as a way to seek medical advice. This advice can be hypothetically free, and there is typically a surplus of information on specific medical topics. According to the Kaiser Family Foundation (KFF), the average annual cost for health insurance in 2024 for a single American is approximately $9000, and for a household it is approximately $26,000 (Section 1, [8]). The KFF analysis of National Health Expenditure also shows that in 2024, the average single coverage and family premiums increased by 6% and 7%, respectively, thus increasing the cost of health insurance for the average American regardless of economic class [8]. Individuals and families from low-income households are often not able to afford high-quality health insurance, and internet searches may be a perceived viable option instead of expensive copays at doctors’ offices.

#### 1.1.2. Medical Deserts

Medical deserts refer to geographic areas where there is limited access to healthcare and medical information [9]. Both rural and urban areas can suffer from this phenomenon. Medical deserts stem from a lack of providers, long distances from providers, or even extended waiting times for appointments. Medical deserts also arise from the lack of specialty providers, which often force individuals to make strenuous travels for specific medical attention. Because of this, many people have turned to the internet for accessible health information.

#### 1.1.3. Language and Cultural Barriers

The public also turns to the internet due to language barriers that persist in the medical system [10,11]. When patients cannot communicate with their physicians, it is an unreasonable assumption that they will have the verbal skills to understand medical jargon to effectively and accurately translate the meanings between English and their primary language. Due to not being able to speak with their doctor, it is not unjustified that a patient will look for outside resources.

Despite the need for doctors to provide satisfactory and adequate care for their patients when language barriers are not easily overcome, it can lead to the spread of misinformation and, in more extreme cases, the distrust of a physician and trauma associated with unfamiliar procedures that were not explained in full. Thus, it is up to the medical system to provide greater access to platforms for translational services or provide human translators to assist patients in understanding their care plan and providing them with the autonomy to make informed decisions about their health on what kinds of care they will receive. Empowering patients and taking the time to communicate with them could greatly reduce the revolving fear of medical professionals in marginalized communities in America and inspire change in what public health and wellness can accomplish.

Furthermore, the internet protects them from potential discrimination or racial, sexual, or otherwise unethical treatment practices based on outdated information and bigotry [12]. Some groups of individuals, particularly women, minorities, and those with disabilities [13], may feel that medical professionals do not listen to them or take them seriously. This inequitable treatment can lead to adverse health outcomes. Individuals of those groups may take to the internet to arm themselves with information so that they can advocate for their health in an attempt to prevent those adverse health outcomes.

### 1.2. What Are the Costs of Poor or False Information?

While there are some clear advantages to turning to the internet for medical advice, there are also some drawbacks [14]. Internet sources can sow mistrust, which is widely appreciated in the realm of politics [15] but is increasingly appreciated in the realm of women’s health [16]. These could range from products sold to treat an ailment but that are unverified in their efficacy to the proliferation of fad or popular treatments that simply do not have clinical value, such as putting butter on a burn [17]. Poor or false information may also exacerbate or worsen a patient’s condition. An example of this is lancing a cyst at home, which often results in infection and return of the cyst. In this case, a patient would have a better prognosis if they had seen a doctor rather than trying to treat themselves at home. Similarly, patients may diagnose themselves incorrectly based on information they find online. Many physical disorders or illnesses have similar and/or overlapping symptoms, in which case testing provided by medical professionals can be crucial in accurate diagnosis. The information online may also not be up-to-date, giving patients possibly inaccurate or incomplete information. Often, the results shown by search engines are determined by the number of views, not by the most recent or quality information. If one tries to circumvent this issue by sorting search results by recency, they are likely to receive a large amount of unrelated or low-quality results.

Another prominent con to retrieving medical information from the internet is that it reinforces skepticism of educated opinions [16,18]. There are various reasons why someone may become skeptical of professional medical care, and internet sources can serve to reinforce that skepticism. If someone finds a particular (perhaps not medically recognized) “condition” online that would explain all of their symptoms, but their physician did not consider or address the possibility of that condition, the patient may feel as if their physician is not competent or truly considering their experiences [19,20]. This reinforces preconceived notions by the patient that their physician is incompetent and may encourage them to turn to the internet should they need help again. Even without initial distrust, the internet excels at generating interaction and aims to keep people engaged, which leads to the creation of echo chambers. Once a patient engages with material discrediting current medical practices or experts, even if the initial skeptical information is accurate, they will be more likely to encounter resources that reinforce distrust of experts and to encounter misinformation at the algorithmic extremes that sway their perception. Additionally, the internet may contribute to the Dunning-Kreuger effect [21], where those seeking medical information online may learn enough to think they are educated enough to self-diagnose, or to influence others, but not enough to understand where they are wrong or what they do not yet know [22]. Despite all of these cons, internet searches will undoubtedly continue for medical information retrieval. Our work aims to highlight trends from systematic searches of specific medical topics, particularly related to women’s health concerns.

## 2. Materials and Methods

### 2.1. Topic Determination

The project was developed as a team science exercise during a course on Nutrient Biology (New Mexico Institute of Mining and Technology, Socorro, NM, USA; BIOL 4089/5089). Students were all women pursuing degrees in Biology and Earth Science, with extensive internet search acumen developed from coursework and personal experience. We (students and professor) devoted ~5 h to discussing women’s health topics prior to searching, defining search criteria, and developing a scoring system. These discussions led to a list of 12, non-cancer health topics particular to women’s health associated with human cis-gender female biology. Considerations of transgender health were discussed, with the consensus decision that those issues are scientifically relevant but deserving of a separate analysis not included here.

### 2.2. Search Protocol

After agreeing on search terms, we experimented with settings in the Advanced Search feature in Google (www.google.com) and collectively agreed to the following settings: Language (English); search terms appearing in the “text” of the page; ANY of the terms “woman”, “women”, “female”; All terms when using a single topic from list above with the addition of the word “nutrient”. To standardize data collection among investigators, all results from the first 5 pages of results were collected. Search result URLs were followed, where a suite of data were gathered (variables in Table 1) and entered into a shared database (Table S1). Definitions for each variable (Table 1) were articulated following a 1-week trial period and further group discussion. Variables were defined to minimize subjectivity across investigators, clarify the reporting of results, and standardize data collection.

### 2.3. Scoring Metric

The scoring metric was developed to allow for mean and variation (standard deviation, SD; standard error, SE) to be calculated from each topic, and compare among topics, and answer how much variation in quality is likely to be encountered across categories of women’s health issues. We report both variation metrics as SD encompasses the variation in the data set, while SE scales for sample size variation among categorical variables. When searching topics using the same criteria:

1. Are some topics more likely to result in results for pages with scientifically verifiable information?
2. Does the quality vary between topics?

Peer-reviewed journal articles were included in the database if encountered in the searches but were removed before statistical analysis. The justification for removing those sources was that it is possible the Google algorithm included those sources disproportionately for our group of college students and a professor who regularly searches for academic articles. We also assume those sources are consulted less frequently by lay audiences searching for health information (Smith citation).

Scores were based on six binary (presence/absence) attributes of each web page evaluated. These were: Author (name present/absent), author credentials given, reviewer, reviewer credentials, sources listed, peer-reviewed sources listed. A score of 1 was given if the attribute was present, and 0 if absent. The total number of references cited on a webpage, as well as the number of those that were peer-reviewed (Table 1) were recorded, but for scoring purposes, a 1 or 0 was assigned if there were or were not references and peer-reviewed references, respectively. Potential scores thus ranged from 0 to 6.

We performed a simple validation experiment via anonymous surveys sent to students at our institution (New Mexico Tech), a predominantly STEM-focused public university. Using the final scores from the search result webpages, a single website from each score was selected at random using the RAND() function in Microsoft Excel to assign a random variable as an identifier to each URL, then sorting by that variable and selecting the first article in a given score category. Webpages with scores of 0 or 6 were excluded from the validation experiment. Following institutional review, a survey was sent to the “all student” email list, and recipients were directed to a web survey that asked participants to give a score of 1–5 to each of the 5 random (but previously scored) web pages, without repeating a score. Participants were given minimal information about the project and had no indication the pages had already been assigned scores. Survey results were collected anonymously by having responses routed to a spreadsheet, and no personally identifiable data were collected from participants.

### 2.4. Statistical Analysis

**Figure 1:**
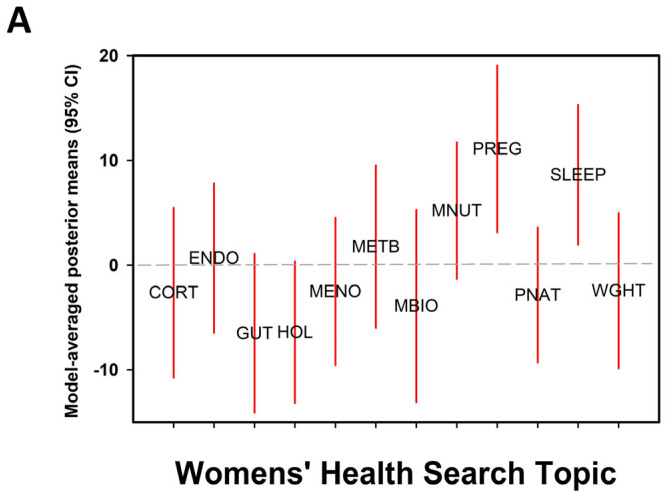

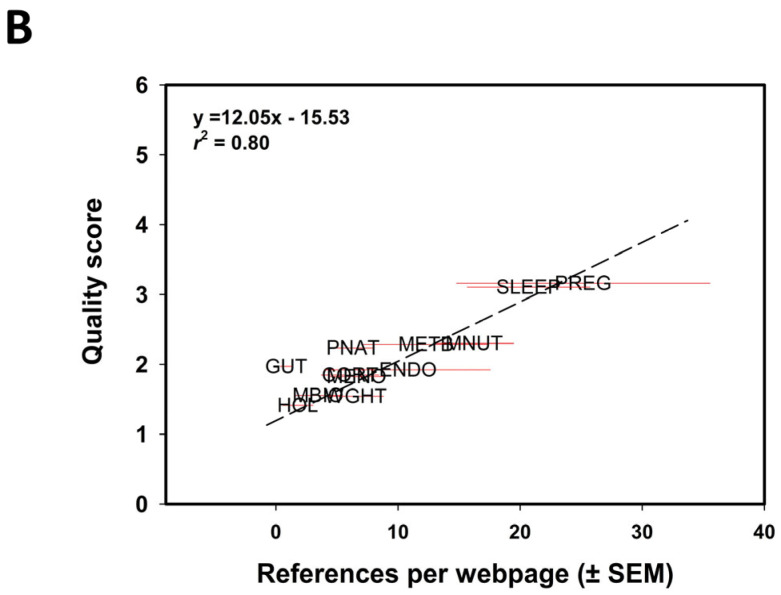
(**A**) Model-averaged posterior mean values from Bayesian linear modeling for quality scores resulting from a systematic internet search of non-peer-reviewed articles across 12 women’s healthcare topics (see Table 2 for definitions). Values for X axis are represented by the symbols at mean. Category abbreviations are mean values, and red bars denote 95% Credible Intervals; interpretation is that when 95% CI does not overlap zero it is a significant result. (**B**) Linear model relationship between references provided per category of women’s healthcare topic and quality score. Category abbreviations represent mean values, and red bars denote 95% Credible Intervals.

Differences in mean scores within each health topic and the mean number of sources per evaluated webpage were evaluated by calculating Bayes Factors; response variables (mean score, number of sources) for each topic were compared to a null model of no difference across topics (y ~ category + error). Output plotted in Figure 1A are the posterior means generated from model evaluation. Equal prior weight was given to each potential model. Variance inequality was tested via Levene’s test, and normality was assessed using quartile-quartile plots. Correlation analysis was used to test the strength of the association between individual scores per website and the number of sources cited per website. Because only the presence or absence of sources was considered in the score calculation, the number of sources is independent of score, and justifies correlation analysis. Statistical analyses were conducted in the open-source software package JASP (version 0.19.2 [23], 2024).

**Table 2:** Search terms chosen, and their rationale for inclusion, used to evaluate information quality of internet sources reporting women’s health topics.

Search Term	Rationale for Term
Cortisol	(CORT) Natural steroid implicated in stress response
Endometriosis	(ENDO) Poorly understood but impacts ~20% of women
Gut	(GUT) Area of body prone to malady, increasing appreciation for its role in overall health
Holistic	(HOLI) Term often applied to alternative, non-Western health strategies
Menopause	(MENO) Characteristic life cycle stage prone to persistent societal myths
Metabolism	(METB) Key feature of biological vitality
Microbiome	(MBIO) Recent appreciation for role of microorganisms in human health
Micronutrients	(MNUT) Disproportionately important for metabolic vigor, enzyme function
Pregnancy	(PREG) Life cycle stage with significant physiological and mental stress
Prenatal	(PNAT) Specific considerations within pregnancy
Sleep	(SLEEP) Necessary for proper body function
Weight	(WGHT) Specifically interested in weight “loss”; all terms are single word

## 3. Results

Our queries following the protocol outlined in the Methods (see above, Section 2) resulted in 557 unique records/URLs (Table S1). After excluding results from academic journal articles, we collected data from 442 websites to calculate quality scores. The categories holistic (n = 46), pregnancy (n = 31) and prenatal (n = 51) were the only groups to not have a zero (0) score. However, gut (n = 32) and microbiome (n = 18) articles only had a high score of 5 across those evaluated, and the highest score for holistic articles was no higher than 4. The lowest variation in article score was for holistic (SD = 0.81; SEM = 0.12), and the highest variation for scores was in the category cortisol (SD = 2.07; SEM = 0.41). 

There was substantial support for differences in scores across the range of search topics (P(M|Data) = 0.99, BF_M_ = 711.58; Figure 1A). The topics *pregnancy* and *sleep* were the two groupings with posterior confidence intervals greater than the group mean and credible intervals not overlapping 0 (pregnancy, 0.792 CI = 0.252 to 1.361; sleep, 0.821 CI = 0.353 to 1.299). The posterior mean for the search topic “holistic” was significantly lower than the group mean (−0.568 CI −1.042 to −0.17). 

The number of references per article was highly variable across health topic categories (Figure 1B). The lowest mean reference per article was the category “gut” at 0.84, while the highest was the category “pregnancy” with 25.16 mean citations per article. The number of sources/citations per article significantly correlated with quality scores (full data set, Pearson correlation; *R* = 0.40, *p* < 0.001). Considering only the mean number of references as a predictor for quality score increased the strength of association considerably (*R* = 0.89, *p* < 0.001; Figure 1B).

## 4. Discussion

The internet will continue to be part of daily experiences for most humans, in any foreseeable future. Just like important discussions about AI are prevalent across the media landscape, discussions and policies regarding the internet at large must also continue. Our increasing reliance on AI tools for web searches exacerbates the need for internet literacy so that the output generated by these tools can be adequately interpreted, especially in the realm of human health decisions.

Our results suggest there is significant variation in the quality of easily accessible women’s health information via internet searches, which varies by topic. Our group’s thoughtful discussion about what women’s health topics were likely to be most searched suggests which ones need to be rigorously interrogated for medical quality.

Some topics affect the majority of women and may therefore be more widely studied than other topics. Increased searches on specific topics could result in more available information and more citations, which in our scoring scheme would result in a higher score. In fact, the two topics with the most significant confidence and credible intervals were pregnancy and sleep, which hold implications even for non-female people. Pregnancy health impacts babies regardless of sex, and sleep is a requirement of every human being, so the wide applicability of these subjects, even outside of women’s health, could contribute to higher scores in our survey of women’s health issues. Ultimately, a more widely studied topic could be more inclined to a higher score due to the greater amount of research available for citation.

In contrast, some topics (e.g., *holistic*, *gut*) may be popular “buzzwords” that receive large amounts of media attention and high internet traffic but that lack corresponding scientific and medical research. These terms may be too general for scientific inquiry, like “holistic”, or they may be upcoming areas of research that have a smaller or unestablished knowledge base, such as “microbiome”. In both cases, there may be fewer scientific or medical articles on that particular term, leading to a reduced number of references and reduced credibility for popular articles about those topics. This highlights a two-fold problem, one being the reliance on popular topics or buzzwords, which may have limited scientific bearing, and the other being that some highly influential topics in the lives of lay people may not receive adequate attention in the scientific and medical communities.

The general trends uncovered in our survey of women’s health topic information shed light on internet literacy, especially in the context of human health decisions. A lack of reputable information for some search terms could indicate that there is a lack of research about those terms. Locating those terms may indicate corresponding gaps in current knowledge, especially within the United States healthcare system, that could be addressed. In one sense, a lack of reputable articles or sources surrounding a particular topic may be an untapped source of communication between lay people and research communities. Following internet search results with tools such as Google Trends can reveal interest in specific issues requiring attention by the scientific community; at the same time, this highlights areas where scientific communication to the laypeople about existing knowledge can be improved. Expanding this work to non-English-language websites would also be a valuable extension. While identifying these gaps in knowledge could be beneficial for directing future scientific research and communication, it does not address the immediate needs of a patient seeking health information online. To this end, an increase in media literacy among the general public is necessary, especially in the age of increasing reliance on AI tools. Media literacy could assist those information seekers in finding the most reliable information online and interpreting it adequately. Increasing awareness of user tools such as advanced search where a patient has more immediate and understandable control, opposed to an AI tool with unknown decision structure, could be extremely beneficial to patients wishing to take more control of their medical information intake.

## 5. Conclusions

A purposeful, systematic internet search of 12 critical women’s health topics suggests that scrutiny is necessary when this information is obtained by a typical internet user. Future work should include review by medical professionals based on their interaction with patients who self-report what they know or think about a condition they present and respect patients’ own internet searching.

## Figures and Tables

**Table 1:** Definitions of categories of webpage attributes used to compile the data set for quality assessment. These attributes are found as column headers in the data provided in Table S1.

Webpage Attribute/Data Category	Definition
Author	A name associated with the writing of the article and held accountable for the content
Reviewed	Explicitly stated that the page content was reviewed prior to website submission
Credential	Listed degree or other professional certification *
Reference List	Citations are formally listed explicitly at the end; not including hyperlinks within the text
Peer Reviewed	Evaluation of scientific, academic, or professional work by others working in the same field (Oxford Languages)
Aim	Article’s intent for use after publication ^†^
Recommendation	Communicated a clear course of action regarding the topic of the article.
Explanation	Description/definition of the category in an unbiased manner.
Page Suffix	The ending of the website URL.
Number of Search Page	The page in which the URL was found on the Google search engine results
Country of Origin	Location of the website’s home base.
Source Ratio	The proportion of references that were peer-reviewed relative to the total ^‡^
Website Description	Category into which the website fell: sales, scientific, medical, organizational, popular, blog
Sales	Website that is purchase-focused with a link or option to purchase product
Academic Journal	Peer-reviewed, primary literature that is research-oriented and published in a journal of empirical research. ^§^
Medical	Associated with a provider of medical training or service. Examples include hospital, medical school, medical practice, or provider
Organizational	Nonprimary; includes charities, universities, and government resources.
Blog	A singular person or group listing their personal experiences or preferences ^¶^
Popular	None of the Above website descriptions.

* Used for both authors and reviewers, truthfulness of the professional/academic certification was assumed; ^†^ Includes commercialized products; ^‡^ Ratio = number peer-reviewed references/total number references listed, peer-reviewed references were determined by following citation to the originally cited journal or by having a DOI/PubMed ID listed. ^§^ For the sake of analyzing popular articles, scientific articles were excluded from the data set when giving the survey (fix wording). ^¶^ If the website listed “blog” explicitly, the website was categorized as such.

## Data Availability

Data are available to recapitulate our statistical results in Table S1.

## Supplementary Materials

The following supporting information can be downloaded at: https://www.mdpi.com/article/10.3390/healthcare13192537/s1, Table S1: Variation in quality of women’s health topic information from systematic internet searches, data set.
